# Much More than M1 and M2 Macrophages, There are also CD169^+^ and TCR^+^ Macrophages

**DOI:** 10.3389/fimmu.2015.00263

**Published:** 2015-05-26

**Authors:** Leslie Chávez-Galán, Maria L. Olleros, Dominique Vesin, Irene Garcia

**Affiliations:** ^1^Department of Pathology and Immunology, Faculty of Medicine, Centre Medical Universitaire (CMU), University of Geneva, Geneva, Switzerland; ^2^Laboratory of Integrative Immunology, National Institute of Respiratory Diseases Ismael Cosio Villegas, Mexico City, Mexico

**Keywords:** monocytes, macrophages, M1/M2 polarization, TAM generation, pathologies

## Abstract

Monocytes are considered to be precursor cells of the mononuclear phagocytic system, and macrophages are one of the leading members of this cellular system. Macrophages play highly diverse roles in maintaining an organism’s integrity by either directly participating in pathogen elimination or repairing tissue under sterile inflammatory conditions. There are different subpopulations of macrophages and each one has its own characteristics and functions. In this review, we summarize present knowledge on the polarization of macrophages that allows the generation of subpopulations called classically activated macrophages or M1 and alternative activated macrophages or M2. Furthermore, there are macrophages that their origin and characterization still remain unclear but have been involved as main players in some human pathologies. Thus, we also review three other categories of macrophages: tumor-associated macrophages, CD169^+^ macrophages, and the recently named TCR^+^ macrophages. Based on the literature, we provide information on the molecular characterization of these macrophage subpopulations and their specific involvement in several human pathologies such as cancer, infectious diseases, obesity, and asthma. The refined characterization of the macrophage subpopulations can be useful in designing new strategies, supplementing those already established for the treatment of diseases using macrophages as a therapeutic target.

## Introduction

The professional phagocytic cells have an efficient ability to ingest particles that have penetrated our innate immunity barriers. This function provides us protection against what would be potentially hazardous to health, whether origin pathogen or not. The monocytes are considered to be precursor cells of the mononuclear phagocytic system that comprises monocytes, macrophages, and dendritic cells (DCs). However, DC population may also originate directly from the DC precursor as revised by Collin et al. (1).

The existence of this cellular network as part of the immune system is required to remove microorganisms through their phagocytic activity and the subsequent induction of immune responses mediated by T cells. But their presence is not only limited to counteracting pathogens with their antimicrobial activity but are also fundamental in repairing tissue under sterile inflammatory situations (2–4).

Thus, macrophage function is not limited to phagocytosis and degradation of pathogens, these cells are also able to discriminate self from non-self, aspect that emphasizes their importance in the development of some tissue. For example, using a murine model carrying a loss-of-function in the gene encoding the transcription factor PU.1 (regulating myeloid protein expression), there was a reduction in the number of vessel intersection was observed leading to abnormal vasculature process and suggesting that macrophage contact with vessel junctions are required to promote vessel fusion (5, 6).

The current macrophage nomenclature is complex and sometimes leads to confusion due to versatile cells, and, as it will be discussed, macrophages possess functional plasticity mediated by microenvironment signals. In this review, we give an overview on the two main macrophage subsets, named according to stimuli inducing their polarization and cytokine profile that they deliver: (a) classically activated macrophages (M1 macrophages) and (b) alternatively activated macrophages (M2 macrophages). We included a discussion on three other subpopulations of monocyte-derived macrophages: (c) tumor-associated macrophages (TAM), one subpopulation involved in cancer; (d) CD169^+^ macrophages; a subpopulation found in lymphoid organs and implicated in immune tolerance and antigen presentation; and (e) one macrophage subpopulation, recently described as T cell receptor positive (TCR^+^) macrophages. Despite the limited information about CD169^+^ and TCR^+^ macrophages, we include them because there are accumulating data showing their role under specific conditions. We describe experimental evidence relative to phenotypic heterogeneity and effector functions and add a short description of the relationship between macrophage subsets and major pathologies where they have been involved.

## Precursor Cell Origin of Macrophages

Monocytes, defined as circulating blood cells, are a population of mononuclear leukocytes, morphologically and phenotypically heterogeneous that constitute approximately 10% of peripheral blood cells in humans. In murine model, it was that blood monocytes are produced by bone marrow (BM) from macrophage/dendritic cell progenitor (MDP) cells. MDP lacks the ability to differentiate into granulocytic, megakaryocytic, lymphoid cells, and are considered as the direct precursor of monocytes from BM and consequently of blood monocytes (7, 8). It has been established that CCR2 and the chemokine chemoattractant protein 3 (MCP-3) are critical for BM monocyte mobilization and homeostasis maintenance (9). Under specific circumstances, the egress of monocytes from blood to inflamed tissue is dependent on both CCR2 and CX3 chemokine receptor 1 (CX3CR1) (10). However, it remains to be determined if these molecules are necessary for the migration of monocytes from BM to blood and finally to tissue, and, if they are also responsible for the recirculation of monocytes through the BM.

In 2003, Geissman et al. identified two populations of murine monocytes: a short-lived subset phenotypically described as Ly6C^+^CX_3_CR1^lo^CCR2^+^CD62L^+^ and found in inflamed tissue, and a second subset with a longer half-life, phenotypically shown as Ly6C^lo^CX_3_CR1^hi^CCR2^−^CD62L^−^ and observed in non-inflamed tissues (11). These subsets of murine monocytes can be compared with the two subpopulations of human monocytes already described nearly 30 years ago (12). Their main characteristics were related to different surface expression markers (CD14, CD16) and their distinct ability to deliver pro-inflammatory cytokines such as tumor necrosis factor alpha (TNF-α or TNF). Currently, these populations are called ‘classical’ (CD14^++^CD16^−^) and non-classical (CD14^+^CD16^++^) monocyte subsets and these are synonymous of CD62L^+^ and CD62L^−^ murine subsets, respectively (13, 14).

**Figure 1:**
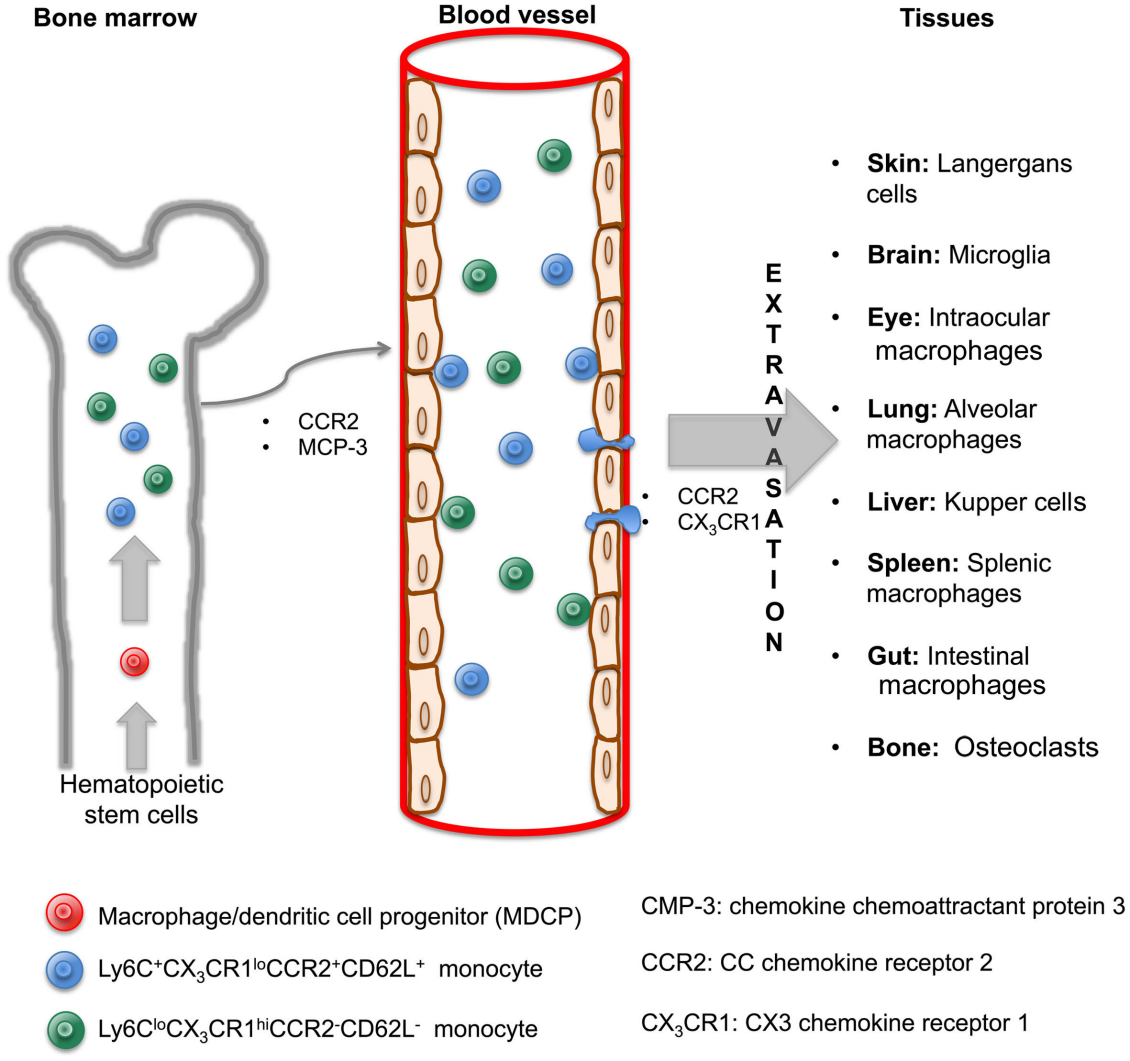
**Monocytes are generated in the bone marrow and mobilized to blood and tissue through cytokine signaling to originate macrophage subsets**. In the bone marrow, hematopoietic stem cells give rise to MDCP, which divides into two subsets of monocytes, Ly6C^+^CX3CR1^lo^CCR2^+^CD62L^+^ and Ly6C^lo^CX3CR1^hi^CCR2^−^CD62L^−^. Both are mobilized to blood through CCR2 and MCP-3 signaling. Once inside the blood vessels, monocytes are able to leave by extravasation and arrive to tissues via CCR2 and CX3CR1 signaling. A macrophage will originate in a tissue-dependent manner and with its own characteristic and name.

Hence, once monocytes are recruited from the BM to circulating blood, they can migrate to various tissues of the body and differentiate into macrophages and subsequently become macrophages with their own phenotypic and functional characteristics in a tissue-dependent manner. According to the specific anatomical site where they are recruited, they are differently named as alveolar macrophages in the lungs, microglia in the central nervous system (CNS), and Kupffer cells in the liver (Figure 1). Recent literature has reviewed macrophage populations and considered the large diversity in phenotype and functions according to their anatomic localization. Italiani and Boraschi have recently reviewed human and murine monocyte subsets and the fate of monocyte/macrophage populations and functions in the main body tissues during steady state and inflammatory conditions (15). Dey et al. provided us an analysis about polarization, ontogeny, and plasticity of tissue macrophages inside a context of treatment of acute and chronic inflammatory diseases (15, 16). Finally, Gordon et al. have summarized the heterogeneity, functionality, and interactions of macrophage populations within body tissues (17).

In this review, we consider blood monocytes as tissue-macrophage progenitors, because the major fraction of macrophages originates from blood-borne monocytes. However, it is important to note that there are tissue-resident macrophages that are not derived from monocytes, and their self-renewal, proliferation, origin, and mechanisms of replacement have not been totally elucidated. In 1989, the presence of macrophages in the mouse yolk sac at day 9 of gestation was reported, suggesting that part of the macrophages may exist before promonocyte and monocyte development (18, 19). In the CNS, a murine model of microgliosis has illustrated that microglia can be functionally maintained independently of BM-derived progenitors (20). By contrast, there is also experimental evidence proving that microglia is derived from hematopoietic cells. Inhibition of monocyte recruitment to the CNS prevented experimental autoimmune encephalitis, suggesting that within the CNS, macrophages can originate from both dependent and independent blood monocytes (21, 22). Recently, Davies et al. reported an extensive analysis confirming the co-existence of macrophages of dependent and independent blood monocyte-derived origin in several tissues including brain, spleen, and lung, and defined cellular phenotypes and functions of these tissue-resident macrophages (23). How the resident macrophage population is self-renewing is still an open question. Colony-stimulating factor 1 (CSF-1) was proposed as a protein necessary to regulate the number of tissue-resident macrophages (24); nevertheless, it has been shown that interleukin-4 (IL-4) plays a major role in favoring the local proliferation of macrophages in parasitic infections. Nevertheless, IL-4-mediated proliferation seems to be independent of CSF-1 (25, 26).

## Origin of M1 and M2 Macrophage Terminology

The concepts M1 and M2 oversimplify the pattern of macrophages because, as we will discuss, the combinatorial spectrum of these cellular populations is extremely large. The first classification of macrophages was adopted from the work on T helper cell polarization. Two main lymphocytic subpopulations were characterized with diametrically contrasting functions and categorized according to patters of cytokines they deliver: T helper cells type 1 (T_H_1) and type 2 (T_H_2) (27).

From early 1980s, Nathan et al. identified that interferon-gamma (IFN-γ) stimulating the peroxide-releasing capacity of macrophages, contributes to macrophage ability to kill intracellular pathogens (28). Posteriorly, IL-4, an anti-inflammatory cytokine, was identified and shown to induce a different macrophage gene expression compared with IFN-γ dependent (29). However, it was only in 2000 when Mills et al. proposed the M1–M2 terminology. These authors used T lymphocytes from two mouse strains, one with a background producing mainly IFN-γ, which activates macrophages to generate nitric oxide (NO) from arginine versus mice which T lymphocytes producing IL-4 and TGF-β1, and generating ornithine from arginine. This elegant study showed that, in a strain-dependent manner, macrophages express distinct metabolic programs and their responses influence inflammatory reactions in opposite way (30).

Recently, Murray et al. proposed a nomenclature and experimental guideline for generating *in vitro* M1 and M2 subpopulations, with the objective to obtain data reproducibility across laboratories (31). In fact, the existence of this guideline notes the relevance to study M1/M2 paradigm as a useful network, which plays different roles inside immune responses.

## Classically Activated Macrophages (M1 Macrophages)

M1 macrophages are defined as macrophages that produce pro-inflammatory cytokines, mediate resistance to pathogens, and exhibit strong microbicidal properties, but these also contribute to tissue destruction. Classical activation of macrophages occurs when the cell receive stimuli such as: (1) IFN-γ, mainly secreted by other cell types (T_H_1 cells, cytotoxic T cells, and NK cells); (2) lipopolysaccharide (LPS), a component of the outer membrane of Gram-negative bacteria; and (3) granulocyte-macrophage colony-stimulating factor (GM-CSF) that stimulates the production of pro-inflammatory cytokines (32–34).

M1 macrophages are characterized by an elevated ability to secrete cytokines such as IL-1β, TNF, IL-12, and IL-18; phenotypically, they express high levels of main histocompatibility complex class II (MHC-II), CD68 marker, and CD80 and CD86 costimulatory molecules. Recently, it has been shown that M1 macrophages up-regulate the expression of intracellular protein called suppressor of cytokine signaling 3 (SOCS3), activate the inducible nitric oxide synthase (NOS2 or iNOS) generating NO. Hence, M1 macrophages, under specific conditions, exacerbate inflammatory processes that can be detrimental to health (35–37). However, these macrophages also have the ability to phagocyte large numbers of pathogens and can kill intracellular bacteria. When macrophages are under classical activation conditions, they initiate microbicidal mechanisms by the synthesis of NO, the restriction of iron or nutrients for microorganisms and acidification of the phagosome (38–40).

At present, the pathway that regulates the macrophage polarization is not fully understood, but there are several molecules implicated in this process. For instance, members of the family of interferon regulatory factor (IRF), signal transducers and activators of transcription (STAT), and SOCSs. In 1990s, STAT1, a 91-kDa cytoplasmic protein, was shown to be crucial for M1 macrophage polarization (41, 42). STAT1 can form homodimers or heterodimers (STAT1–STAT2) that bind to interferon-stimulated response elements (ISREs) and members of the IRF can also bind to ISRE sequences. In 2011, Krausgruber et al. showed that IRF5 is a critical protein for M1 macrophage polarization. Both GM-CSF and IFN-γ stimuli induce IRF5 expression that directly activate 20 M1-specific genes and inhibit 19 M2-specific genes encoding cytokines (43).

Lipopolysaccharide stimulus generates M1 macrophages through interaction with its receptor, TLR-4, by inducing phosphorylation of both STAT1α and STAT1β. This pathway is MyD88-independent but is toll/IL-1R motif-dependent (44). A contribution from Bruton’s tyrosine kinase (Btk) is possible at this level since Btk is required downstream of TLR-4 for optimal phosphorylation of STAT1, and its absence exacerbates M2 recruitment under allergic inflammation conditions (45). Recently, Eun et al. showed that the P2Y(2) receptor (P2Y(2)R), a G-protein-coupled receptor, is up-regulated in response to LPS and facilitates the release of ATP, thus, P2Y(2)R increases NOS2–NO levels, which is a signature of M1 polarization (46). Arnold et al. reported experimental evidence supporting the hypothesis that up-regulation of SOCS3 is essential for an effective M1 macrophage activation. Indeed, SOCS3 controls activation and translocation of nuclear factor-κB (NF-κB) and activity of phosphatidylinositol 3-kinase (PI3K), favoring NO production (37).

Finally, it has been shown that in an autocrine or paracrine manner, Activin A, a growth and differentiation factor of the TGF-β superfamily, promotes the expression of M1 markers and down-regulates the production of IL-10 probably leading to M1 polarization (47, 48).

## Autoimmune Diseases-Related M1 Macrophages

Autoimmune diseases are frequently associated with inflammatory processes. Here, we briefly describe experimental evidence showing a relationship between molecules previously described that support M1 polarization and the pathophysiology of autoimmune diseases.

The inflammatory bowel disease (IBD) is characterized by a chronic recurrent inflammation of the gastrointestinal tract. In both, murine model and biopsies of IBD patients, increase of SOCS3 expression has been observed, which was correlated with the severity of inflammation. Furthermore, SOCS3 expression has been proposed as a useful marker for cells undergoing acute or chronic inflammation (49–52). In systemic lupus erythematosus (SLE), an autoimmune illness characterized by chronic inflammation, patients displayed elevated levels of IFN-γ and IRF-5 (53). In addition, a risk haplotype of IRF5 has been described in SLE patients associated with the presence of anti-double-stranded DNA antibodies, which preceded clinical symptoms in many individuals (54). In addition, there is evidence that the SLE risk haplotype also influences systemic sclerosis predisposition, which is a fibrotic autoimmune diseases (55).

Multiple sclerosis (MS) is a demyelinating disease of the CNS. The role of macrophages in the CNS appears dual. Initially, it was shown that activated monocytes/macrophages could play a role in the acute exacerbation of MS in patients (56). However, experimental evidence suggested that GM-CSF (M1 inductor) was necessary to induce axonal regeneration (57). Using the experimental autoimmune encephalomyelitis (EAE) murine model, it has been reported that macrophages producing NO played an active role in the inflammation pathogenesis (58, 59). Experimental approaches attempt to reduce CNS inflammation using clodronate liposomes to eliminate infiltrating macrophages as well as G-protein-coupled receptor (GPBAR1), agonist to reduce pro-inflammatory cytokines; mice treated with clodronate liposomes or GPBAR1 agonist exhibited a significant decrease in EAE clinical score (60, 61).

Rheumatoid arthritis (RA) is an autoimmune disease characterized by a chronic inflammation of the synovial membrane that leads to joint destruction. Macrophage number has been correlated with disease activity in RA patients (62). Specifically, it has been shown that STAT1 is a signature in RA synovial fluid macrophages and dependent on autocrine TNF (63). At date, an effective treatment is proposed to block members of Janus kinase (JAK) family, JAK1 and JAK3, since these molecules are necessary to phosphorylated STATs modulating gene expression toward M1 profile (64).

Type 1 diabetes mellitus is an autoimmune disease characterized by depletion of insulin-producing pancreatic beta cells (β-cells) and consequently, high levels of glucose. Under hyperglycemic condition, the generation of reactive oxygen intermediates and apoptosis by IFN-γ/STAT1-dependent pathway is increased (65, 66). In murine model, it has been shown that β-cells increase the expression of CCL2 and allow pro-inflammatory monocyte recruitment in the pancreas and spontaneous development of diabetes (67). It has also been shown that macrophages from diabetic mice have elevated expression of anti-apoptotic proteins as a potential mechanism to promote an attack toward β-cells (68).

## Obesity-Related M1 Macrophages

Obesity has been implicated as the major risk factor in developing diverse diseases such as type 2 diabetes mellitus (69). Below, we discuss diverse evidence showing that obesity has a pro-inflammatory background. Although on a cellular level still remains largely unknown; however, it has been described, both *in vitro* and *in vivo*, that human adipocytes up-regulate of NF-κB-regulated genes, such as CCL2, E-selectin, IL-6, and IL-8, favoring the recruitment of macrophages (70, 71).

Weisberg et al. showed that macrophage numbers were increased in adipose tissue in both mice and obese humans; their percentage correlated positively with their level of obesity, and these cells were responsible for almost all of the TNF and iNOS present in adipose tissue (72). Macrophages isolated from white adipose tissue from lean animals showed hallmarks of polarization toward an alternative activation state. By contrast, obesity induced increasing gene expression of molecules characteristic of M1 macrophages, such as those encoding TNF and NOS2, suggesting that diet-induced obesity leads to a change from M2 to M1 polarization (72, 73). Animal models have shown that a high-fat diet increases systemic and tissue pro-inflammatory cytokines such as bioactive TNF, IL-6, and IL-12, reinforcing the hypothesis that obesity favors a pro-inflammatory microenvironment (74). In agreement with these data, Kawanishi et al. reported that physical activity markedly inhibited TNF and increased CD163 (a M2 marker) mRNA expression in adipose tissue. Thus, it appears likely that exercise might induce the phenotypic switching from M1 macrophages to M2 macrophages in high-fat-diet animals (75).

## Infectious Diseases-Related M1 Macrophages

Pro-inflammatory macrophages are necessary in controlling infectious processes, principally intracellular pathogens such as *Mycobacterium tuberculosis* (*M. tuberculosis*) or *Listeria monocytogenes* (*L. monocytogenes*), causal agents of tuberculosis and listeriosis, respectively. The array of pro-inflammatory and chemoattractant cytokines induced by *M. tuberculosis* infection play a significant role in cell recruitment, granuloma formation and progression, and severity of the disease (76). However, the inductions of pro-inflammatory cytokines and microbicidal macrophage functions are necessary to activate protective host immune responses against mycobacterial infections (77, 78). It has also been proposed in murine model that during the later stages of infection the presence of alternatively active macrophages are increased, impairing NO production, and consequently favoring the intracellular persistence of mycobacteria (79). Leemans et al. reported that macrophages play a dual role during tuberculosis since depletion of non-selective macrophage populations improved the clinical outcome, while depletion of activated macrophages enhanced mycobacterial outgrowth and decreased granuloma number, which is associated with a deficiency of TNF production (80). Mycobacterial proteins suppress functions of M1 macrophages as a mechanism of pathogen evasion. For instance, macrophages infected with a mycobacterial deficient strain of SecA2 produced high levels of TNF, IL-6, and reactive nitrogen intermediate (81), but the PE-PGRS62 protein supported virulence through down-regulating IL-1β and iNOS mRNA levels (82, 83). These data, therefore, indicate that for the adequate control of *M. tuberculosis* infection, the development of a bactericidal mechanism of M1 macrophages is required.

*Listeria monocytogenes* is a foodborne bacteria infecting a majority of organs including liver in which induces recruitment of pro-inflammatory monocytes and formation of micro-abscesses containing among other cells M1 monocyte-derived macrophages. Several chemokines have been shown to play a critical role in monocyte recruitment and infection control such as MCP-1 (also referred as CCL2, the ligand of CCR2), and MCP-3 (84). Mice deficient in CCR2 were unable to clear *L. monocytogenes* infection (85–87). The Notch signaling regulates diverse cellular process and after ligation with its ligand, it is cleaved, translocated to the nucleus, and binds to the DNA-binding protein RBP-J (88). Notch-RBP-J and TLR-4 pathways synergistically induce expression of IRF8 protein that plays a role in activation of M1 macrophage polarization, autophagy, and clearance of *L. monocytogenes* (89, 90). An interesting study by Bleriot et al. has shown that during of *L. monocytogenes* infection, necroptosis of Kupffer cells delivers IL-1β activating hepatocytes to produce the alarmin IL-33 triggering the basophil to produce IL-4 and resulting in a shift of M1 to M2 macrophage phenotype. These M2 macrophages can replace dead Kupffer cells allowing attenuation of the inflammatory process and return to liver homeostasis. This study illustrates that sequential type 1 and type 2 immune responses after *L. monocytogenes* infection enable first host defense and then homeostasis (91).

## Alternatively Activated Macrophages (M2 Macrophages)

Macrophages activated through a pathway opposite to the classical pathway are referred to as M2 or alternative pathway. It has been demonstrated that stimuli such as CSF-1, IL-4, IL-10, TGF-β, and IL-13, fungi and helminth infections, favor M2 subpopulation polarization, delivering IL-10 in high concentrations, and IL-12 in low amounts. M2 macrophages play a central role in responses to parasites, tissue remodeling, angiogenesis, and allergic diseases (25, 92).

Phenotypically, this population is characterized by the expression of the macrophage mannose receptor (MMR), also called CD206. However, in 2013 Jaguin et al. observed no difference in CD206 expression between M1 and M2 macrophages and proposed that specific characteristic of M2 macrophages is the up-regulation of the CD200R membrane glycoprotein (93). CD163 has been suggested as M2 marker, but more recently was shown, in human tissue, that CD163 is M2 macrophage marker only in combination with the transcription factor CMAF, thus CD163 cannot be considered a M2 marker when used as unique marker (94). MGL1 and MGL2, two members of the macrophage galactose-type C-type lectin family, are also expressed in macrophages stimulated upon conditions of alternative activation (95). Response gene to complement 32 (RGC-32) is a cell cycle regulator expressed in many cells including macrophages but not monocytes (96). Recently, it has been shown that absence of RGC-32 does not affect monocyte-macrophage differentiation, however, under M-CSF or IL-4 stimuli, RGC-32 has a relevant role to promote M2 polarization and its level of expression still increases M2 macrophages, proposing this protein to be included as a marker for M2 polarization (97).

Using a murine model, a genetic profile for M2 macrophages has been reported, and among other genes, arginase 1 (*Arg1*), MMR (*Mrc1*), resistin-like molecule α (*FIZZ1*), and chitinase-like protein *Ym1* were shown to be up-regulated (98). In conclusion, to make an adequate phenotypic characterization of macrophage subpopulations, it is important to consider all described markers rather than individual markers, considering that some molecules can be shared by distinct cellular subsets.

The molecular network activated to favor M2 polarization involves distinct members of families already discussed to participate in M1 polarization. On the one hand, Ohmori et al. showed that IL-4 is antagonistic to IFN-γ because it can suppress IFN-γ-stimulated gene transcription and increase the activation of STAT6 (99). Arginase 1 production, a hallmark of M2 macrophages, depends on IL-4 and IL-13, and is a direct consequence of STAT6 activation (100). It has been shown that the NFκB p50 subunit, in the form of homodimers, has a regulatory activity essential for M2 polarization, both *in vitro* and *in vivo* (101). IRF4, a member of IRF family, is involved in M2 polarization and functions as a negative regulator of TLR signaling by association with the MyD88 adaptor molecule (102). Peroxisome proliferator-activated receptor γ (PPARγ), a nuclear receptor with anti-inflammatory properties, has been proposed to enhance the M2 phenotype; however, only native monocytes can be primed by PPARγ, but not M2 resting or M1 macrophages (103). The treatment of human monocytes with bone morphogenetic protein-7 (BMP-7) induces M2 polarization and appears to be mediated by the p-PI3K–Akt–mTOR complex (104). On the other hand, it has been proposed that IL-21 can affect M2 polarization by inhibiting ERK phosphorylation and decreasing iNOS, thus consequently increasing STAT3 phosphorylation (105). The detailed molecular network controlling M2 polarization still appears to be complex and is not fully understood.

## M2 Macrophages in Allergy and Asthma

Allergic asthma is an inflammatory disease of the airway characterized by an increased reactivity to different allergens, and by T_H_2 immune responses expressing IL-4, IL-5, and IL-13, as recently revised by Lambrecht and Hammad (106). Some clinical hallmarks of asthma include increased IgE, eosinophilia, and airway hyperresponsiveness (AHR) (107). At the protein level, asthma pathology is known to depend on IL-4/IL-13 acting through IL-4 receptor-alpha chain (IL-4Rα) and activating the STAT6 pathway (108). At present, exactly how M2 macrophages contribute to this pathology is not completely clear. Evidence using ovalbumin and house dust mites to induce an allergic airway disease in IL-4Rα KO mice, showed a decrease in macrophage Arginase1^+^ and Ym-1^+^ but the clinical pathological features were not reduced. By contrast, the presence of YM1-1^+^ macrophages responding to IL-4/IL-13 increased the severity of allergic lug inflammation as a consequence of M2 macrophage production of CCL11 and CCL24, two important mediators of eosinophil chemotaxis (109, 110). Thus, it is possible that the discrepancy regarding the role of M2 macrophages in asthma physiopathology depends on different factors including the nature of the stimulus and the time of exposure.

## M2 Macrophages in Helminth Parasite Infection

Helminths have subversive immune strategies that allow them to evade the host’s immune response and to establish persistent infections. In 1998, Barner et al. showed that IL-4Rα-deficient mice were extremely susceptible to helminth infections suggesting that T_H_2 responses regulate this type of infection (111). M2 macrophages are necessary for an adequate immune response against parasites; however, the exact mechanism through which M2 macrophages act has not been fully elucidated. It has been proposed that M2 macrophages are an effector cell population impairing parasite health and mobility through the arginase 1 pathway and contributing to expulsion of adult worms (112). Using a model of the *Strongyloides stercoralis* infection, it has been reported that M2 macrophages can kill the parasites but this function depends on the collaboration between neutrophils and the complement system (113). Finally, the role of M2 macrophages during nematode infection has been considered to be not only limited to the elimination of the parasite but rather the T_H_2-type response is essential for controlling acute tissue damage and repair, which is their traditional role (114).

## Tumor-Associated Macrophages

In a strict sense, TAM are not always considered as an additional subset of macrophages because these cells do not exist at steady-state condition but are observed in many tumors. TAM are macrophages associated with a specific pathological context and their specific polarization status has been the object of extensive studies supporting TAM as M2-like macrophages. However, there are also experimental evidence not only proposing TAM as one unique and distinct M2 myeloid population but also sharing M1 and M2 signature polarization (115–117). Switch of M2 macrophages toward M1 phenotype has been proposed as a therapeutic approach (118).

The microenvironment of solid tumors, releasing many chemoattractants, has the ability to induce recruitment of circulating monocytes, which may differentiate into TAM and are frequently found in tumors forming the main inflammatory infiltrate. It has been proposed that TAM can induce angiogenesis, lymphogenesis, stroma remodeling, immune suppression, and metastasis. TAM release different enzymes comprising plasmin, uPA, MMP, and cathepsin B contributing to tumor cell invasiveness and metastasis (119, 120). These cells accumulate in tumors mainly in necrotic regions, and are associated with a poor prognosis (121, 122).

Even if some features of TAM resemble M2 polarization, such as high production of IL-10, the same cells co-express IFN-inducible chemokines; this duality was recently described in freshly isolated TAM from pancreatic ductal adenocarcinoma. Cells exhibited anti- as well as pro-inflammatory properties supporting the idea that macrophages are main players of the epithelial–mesenchymal-transition favoring tumor formation and metastasis (123).

Although TAM show a pattern of M1 and M2 macrophages, it is known that these cells have a transcriptional profile distinct from M1 or M2 macrophages. Considering that there is an “overlap” of molecules between M1/M2/TAM, Table 1 summarizes some of the discrete differences that have been reported on molecules considered as specify marker for every one of these three types of macrophages.

**Table 1:** **Molecules involved in the polarization of M1, M2, and TAM macrophages**.

	M1	M2	TAM
iNOS production	**↑**	−	**+**
MHC-II expression	**↑**	**↓**	**↓**
CD163 expression	−	**+**	**+**
CD200R	−	**+**	−
Macrophage galactase-type C-type lectins	**↓**	**↑**	**↑**
Response to stimulus	IFN-y, LPS, GM-CSF	IL-4, IL-13, M-CSF, helminths	Tumor microenviroment
Arginase 1	−	**↑**	**+**
Cytokine production	IL-18, IL-12, IL-1, TNF	IL-10, IL-12(low)	IL-10, TGF-β, CCL2, CCL5
STAT molecules	STAT1, STAT2	STAT3, STAT6	STAT1
IRF molecules	IRF5	IRF4	**↑**^IRF3^
NFκB participation	p65	p50	p50
PI3K participation	**+**	**+**	**+**
Genes	*Nos2*, *Ciita*, *ll12*	*Arg1*, *Ym1*, *ll10*, *Mcr1*, *Fizz1*	*Ccl2*, *Ccl5*, *ll10*, *CD81*, *H2Eb*

*‘+’ Present. ‘−’ Ausent. **↑** High expression or production. **↓** Low expression or production*.

As yet, it is not completely clear how the cascade of events generating TAM is orchestrated. Resting TAM are characterized by high expression of CCL2, CCL5, and IL-10 and surface molecular markers such as MGL1, Dectin-1, CD81, MHC-II, and scavenger receptor A (121, 124). It has been described that TAM activation pathway enhances IRF3, STAT1, and the release of CCL2, CCL3, CCL5, IL-10, IL-12 (low), as well as other molecules such as PGE2 and epidermal growth factor (124, 125). In a model of TAM derived from murine and human tumors, it has been shown that LPS activation of TAM resulted in defective NF-κB activation as well as inhibition of IL-12p40 promoter transcriptional activity. However, when TAM were cultured in standard conditions (outside the tumor microenvironment), they recovered the capacity to express IL-12 and TNF (126). These data suggest that tumor environment induces TAM to maintain a status of tolerance and consequently this may attenuate macrophage cytokine responses.

There is no precise information on how a monocyte precursor can generate the coexistance of both M1 and M2 markers in the same TAM. Regarding this question, Movahedi et al. reported that the tumor-infiltrating monocyte pool were predominantly Ly6C^+^CX_3_CR1^lo^ and suggested that Ly6C^high^ monocytes were direct precursors of TAM. Then, they subdivided TAM in two groups according to intensity of MHC-II expression and suppression of T cell proliferation: (1) MHC-II^hi^ TAM suppressing proliferation using an iNOS-pathway and (2) MHC-II^low^ TAM suppressing proliferation in an iNOS-independent pathway (127). Recently, Franklin et al. reported experimental evidence supporting the idea that inflammatory monocytes could differentiate into TAM; as a late differentiation event, the integrin receptor VCAM1 was up-regulated, but terminal differentiation was dependent on the transcriptional regulator of Notch signaling, RBPJ (128). Laoui et al. have reviewed TAM subsets in breast cancer defining markers and functions (129).

It is important to note that, as previously discussed, STAT3 is involved in M2 macrophage differentiation; however, in addition, STAT3 also participates in TAM functions. STAT3 is required for expression of DC-SIGN on macrophages that might help tumor progression because these cells releasing IL-10 favor the maintenance of an activated STAT3 in a tumor context (130). In a hepatocellular carcinoma (HCC) model, it was shown that TAM secrete IL-6 activating STAT3 in HCC cells and promoting expansion of cancer stem cells. This group proposed that targeting IL6/STAT3 to inhibit cancer stem cell function has important therapy implications for the treatment of HCC (131). More recently, using the HCC model, a study identified that TAM produced high levels of IL-8, chemokine that induced epithelial–mesenchymal transition and promoted cellular migration by JAK2/STAT3 signaling pathway (132). Thus, these data indicate that STAT3 has a relevant participation in tumor progression, and could be considered as target molecule for anticancer therapy.

*Mycobacterium bovis* bacillus Calmette–Guerin (BCG) has been used for the immunotherapy of bladder carcinoma for more than 30 years. It is clearly established that experimental BCG infections in murine model and in human vaccination induce a Th1 type immune response with activation of IFN-γ and TNF (77). Two strains of BCG, which are currently used for bladder carcinoma immunotherapy, were recently compared and the results showed that BCG Connaught strain provided better clinical outcome and better patient survival. In the same study, comparison of the two BCG strains in a murine model of bladder carcinoma demonstrated that BCG Connaught strain-induced stronger Th1 immune responses suggesting that better BCG efficacy is associated to the activation of Th1 type immunity (133). Attempts to associate BCG treatment with TAM and M1 and M2 are in progress. The efficacy of BCG instillation in non-muscle invasive bladder carcinoma was associated with decrease M2, suggesting that M2 tumor infiltration can be considered as a marker for recurrence of tumors (134). Clinical trial using sequential combination of BCG and mitomycin with the objective to improve bladder cancer immunotherapy and to increase M1 TAM are in development (135).

## CD169^+^ Macrophages

The antigen CD169, or Siglec-1, was originally reported as a marker of one macrophage subpopulation isolated from BM, lymph node, liver, and spleen (136). These cells had the ability to bind red cells. The CD169 molecule is highly expressed by macrophages found within the subcapsular sinus (SCS) and the medulla (M) of lymph nodes (LN) and marginal-zone (MZ) in spleen. So far, there is not sufficient information regarding the signaling pathway and activation of CD169^+^ macrophages as defined for M1 and M2 macrophages (136, 137).

**Figure 2:**
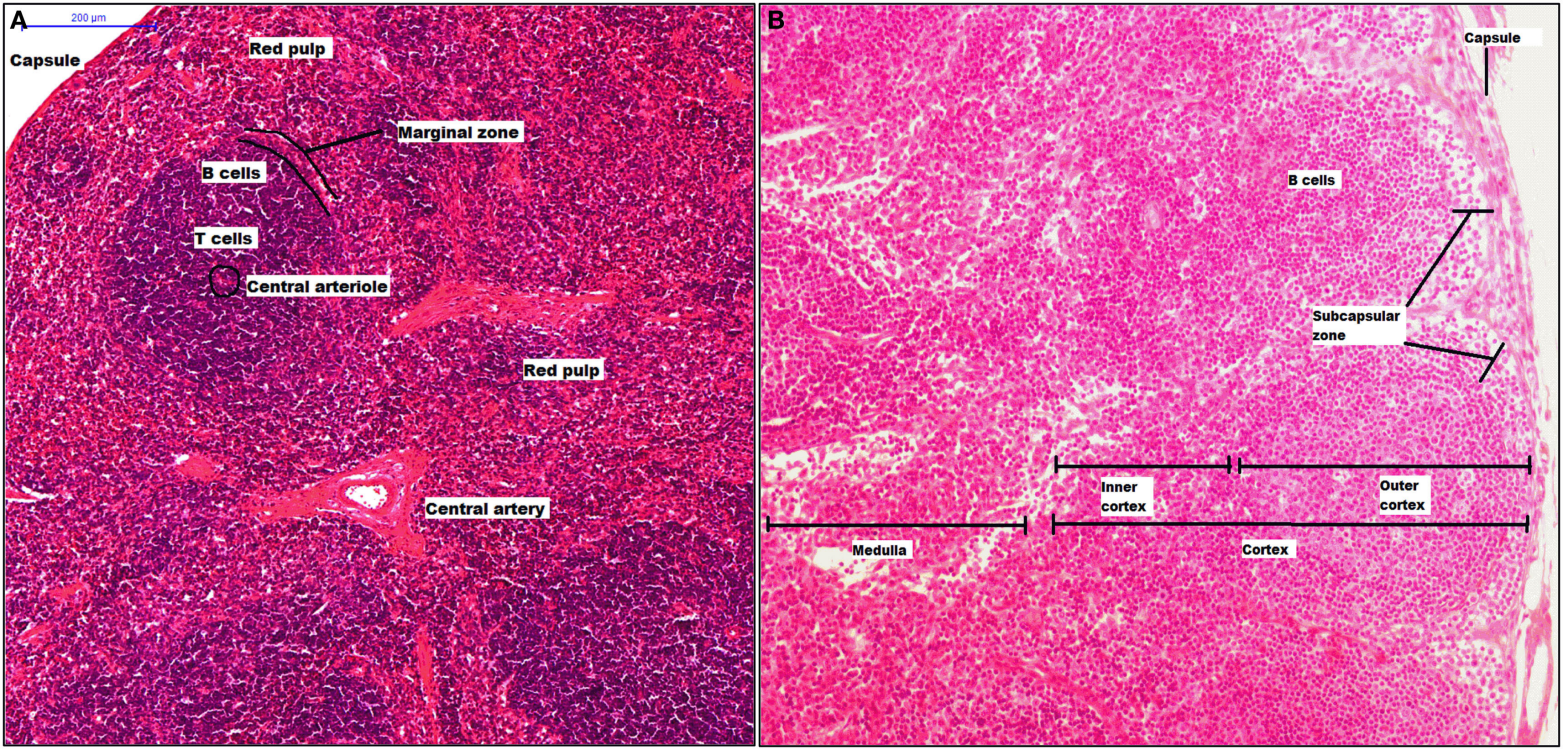
**Structure of spleen and lymph node**. The spleen has two mayor components, white pulp that includes a central arteriole, T and B cells, and red pulp. Between red and white zone, there is a marginal zone where there are CD169^+^ macrophages **(A)**. A lymph node is surrounded by a capsule, and the parenchyma is divided into cortex and medulla. The cortex has two zones: outer and inner zone. The subcapsular sinus and medulla zone contain CD169^+^ macrophages **(B)**.

The spleen has crucial roles in our body as filtering the blood, erythropoiesis, and is the largest secondary lymphoid organ. This organ is structured in compartments, comprising the white pulp that consists of a central arteriole and the T and B cell areas (splenic nodule), the red pulp surrounds the white pulp, and both pulps interact at the MZ (Figure 2A). From 1986, it was described that the murine MZ contains marginal metallophilic cells that are CD169^+^ and also promote adaptive immune response (138, 139).

Lymph nodes are secondary lymphoid organs integrated into the lymphatic system, whose main activities include filtering the lymph, maintaining and producing B cells, and detection of lymph-borne antigens. A LN is surrounded by a capsule, which is underlined with lymphatic endothelial cells forming the SCS. The parenchyma is divided into cortex, which has two zones: the outer cortex (B cell-rich) and the inner cortex (CD4^+^ T cell-rich), and the medulla (Figure 2B) (140). Thus, CD169^+^ macrophages are localized inside the SCS and M and their phenotype characterization remains ambiguous. In murine model, it has been clearly described some molecules as part of CD169^+^ macrophages characterization, the molecules included are CD11b, MHC-II, CD68, CD11c, and F4/80. However, F4/80 is expressed only on some CD169^+^ macrophage subpopulations. It has been proposed that inside of SCS there are CD169^+^ macrophages that are F4/80^−^ while macrophages from the M are F4/80^+^. Finally, SCS macrophages, as splenic CD169^+^ macrophages, require lymphotoxin alpha (LT-α) signaling and can be depleted using clodronate liposomes (141, 142).

Recently, it was reported that CD169^+^ macrophages could be detected in the colon, interestingly, these macrophages are vitamin A dependent for their proper development in contrast to splenic CD169^+^ macrophages, which are LT-α-dependent and express low levels of F4/80 and CD11c (143, 144).

The biological functions of CD169^+^ macrophages are still imprecise. CD169^+^ macrophages do not mediate phagocytosis and are mainly involved in the regulation of the immune system rather than in steady-state hematopoiesis (145). However, as discussed below, their best described functions may be conditioned primarily upon their anatomic localization, and it has been proposed some activity in kidney pathologies and under viral infection.

## CD169^+^ Macrophages and Erythropoiesis

The BM is the main site for adult hematopoiesis where erythroid progenitors develop into red blood cells, a process orchestrated mainly by erythropoietin. Chow et al. demonstrated a new role for the mononuclear phagocytes as erythropoietin-complementary regulators. They found that CD169^+^ macrophages from the BM promoted retention of the hematopoietic stem cells (HSC) by acting on the Nestin^+^ HCS, suggesting that G-CSF signaling in macrophages is sufficient to promote HSC mobilization (146). Posteriorly, it was reported that G-CSF and depletion of CD169^+^ macrophages blocked erythropoiesis in the BM but not in the spleen (147). Supporting the relevant role of CD169^+^ macrophages in erythropoiesis, Falchi et al., using an *in vitro* erythroid stress model, recently reported that dexamethasone (Dex) amplified the number of proerytroblasts (proEry) and maintained proliferation in an indirect form. They showed that Dex promoted the CD169^+^ macrophage maturation and instructed to exert their erythroid function, thus acting as a regulator of stress erythropoiesis (148).

Polycythemia vera (PV) and β-thalasemia (β-T) are diseases associated with an elevated erythropoietic activity. PV is a clonal stem cell disorder with the somatic JAK2^V617^F mutation, whereas β-T is an expansion of the pool of erythroid progenitors. Consistent with previous reports, Ramos et al. have provided experimental evidence suggesting that macrophage depletion in mice with the JAK2^V617F+^ mutation delayed the appearance of PV and proposed the JAK2^V617F^ mutation as responsible for initiating the pathology, but also a stress erythropoiesis macrophage-supporting activity (SEMA) is required for a full manifestation of the erythroid phenotype *in vivo* (149). Together, these results are crucial for the identification of new therapies for hematopoiesis disorders where the strategies may use macrophages from BM as a new target cell.

## CD169^+^ Macrophages and Their Immunological Role

Invariant natural killer T cells (iNKT cells) are a subset of T lymphocytes that express a specific αβ T cell receptor (TCR), Vα14-Jα18 in mice and Vα24-Jα18 in human, co-express molecules of NK cells (CD16, CD56), and recognize lipid antigens presented by CD1d molecules. CD169^+^ macrophages can mediate rapid and long-lasting interactions with iNKT cells after administration of lipid antigens (150). CD169^+^ macrophages can induce a similar robust activation of iNKT cells *in vivo*, using liposomes decorated with glycan (151).

Immunological tolerance is the ability the body has to distinguish between self and foreign, allowing homeostasis maintenance and prevention of autoimmune diseases. Two models propose that CD169^+^ macrophages are necessary to maintain immunological tolerance. The first model suggested that in the splenic MZ, apoptotic cells induce the expression of CCL22 in CD169^+^ macrophages, resulting in rapid follicular accumulation of Tregs and 103^neg^ DC subset. Thus, recruited Tregs could be activated by apoptotic cell antigens when presented by professional antigen presenting cells or constitutively self-antigen presented to maintain tolerogenic stimulation (152). The second model proposed that CD169^+^ macrophages from the lymph node or spleen are responsible for capturing exosomes, cell-derived vesicles that are a potential source of self-antigens, thus suggesting that CD169^+^ macrophages control the access of exosomes to lymphoid organs as a mechanism decreasing the probability of self-antigen responses (153). Although at date, mechanisms favoring generation and maintenance of immunological tolerance have not been totally elucidated, CD169^+^ macrophages can be considered important players.

## CD169^+^ Macrophages in Kidney Diseases

Some kidney diseases have been associated with macrophage accumulation. In a model of anti-glomerular basement membrane (anti-GBM), adoptive transfer of macrophages showed their contribution to renal injury-induced proteinuria and glomerular cell proliferation (154). Posteriorly, CD169^+^ macrophages were found in glomerulonephritis and there was a correlation with proteinuria and histologic damage, however, as CD169 marker is absent from blood monocytes, this work did not identify if a specific factor, within the glomerular microenvironment, induced the ­expression of CD169 on macrophages or if CD169^+^ macrophages were recruited (155).

More recently, using a model of renal ischemia–reperfusion injury (IRI), Karasawa et al. identified a subset of peripheral blood monocytes (inside CX_3_CR1^+^ subset) and kidney-resident macrophages, which were CD169^+^. However, in contrast to anti-GBM model, the depletion of CD169^+^ cells resulted in progressive renal injury by IRI, suggesting that CD69^+^ cells contributed to the suppression or resolution of IRI (156).

## CD169^+^ Macrophages in Their Antiviral Role

NK cells play a pivotal role in viral infection. Garcia et al. have shown that during experimental infection with recombinant modified vaccinia virus Ankara (MVA), viral vector used for vaccine purposes, NK cells accumulated in LN and became activated in an IFN-dependent manner. Indeed, CD169^+^ macrophages from the SCS are the main type I IFN producers and therefore SCS macrophages appear essential for NK cell recruitment (154). A second infectious viral model using vesicular stomatitis virus (VSV) also supported that SCS macrophage can contribute to antiviral immune surveillance (157, 158). With the same model of VSV, other group reported that CD169^+^ macrophages from the MZ were able to capture virus but allowed viral replication even in the presence of type I IFN and also overexpressed Usp18, a potent inhibitor of IFN signaling pathway. The lack of either CD169^+^ cells or *Usp18* led to impaired adaptive immunity against VSV suggesting that enforced viral replication in CD169^+^ macrophages is essential for the induction of an efficient adaptive immune response (159).

It has been shown that increased Siglec-1 expression on human CD14^+^ monocytes in response to human immunodeficient virus (HIV-1) infection, correlated with viral loads that range from undetectable (<50 copies/ml) to unsuppressed (>800,000 RNA copies/ml), proposing that Siglec-1 avidly binds HIV-1 and facilitates virus dissemination. It is possible that sialic acids on the viral envelope facilitated HIV-1 infection of macrophages through interacting with Siglec-1 (160, 161).

## TCR^+^ Macrophages

T cell receptor (TCR), a molecule necessary for antigen recognition with broad antigen specificity, forms a complex with CD3. This complex TCR-CD3 consists of eight chains: two from TCR that are mainly αβ, but occasionally γδ, plus six chains from CD3: δϵ, γϵ, and ζζ. For many years, it has been accepted that TCR expression is exclusive to T cells; however, there are no reports providing explicit or systematic experimental evidence that others leukocytes, different from the T cell lineage are unable to express TCR. Puellmann et al. reported experimental data changing this dogma (162).

In 2006, it was reported that 5–8% of neutrophils in the circulation express TCR-αβ complex, comprising CD3 and individual-specific TCR Vαβ repertoires, the engagement of the CD3-dependent TCR signal in neutrophils inhibited apoptosis and increased IL-8 expression (162, 163). In addition, other ­cellular subsets from polymorphonuclear group were also shown to express TCR. Interestingly, there are eosinophil subsets that are TCRγδ^+^ and TCRαβ^−^, when these cells were activated by CD3 (unspecific stimulus) led to ROS production, eosinophil peroxidase (EPO), eosinophil-derived neurotoxin (EDN), and cytokine release. However, when eosinophils were activated by mycobacterial ligands (specific stimulus), which are efficient to activate T cells TCRγδ^+^, they produced ROS and EPO but released cytokines were not documented. Finally, this subpopulation also had an anti-tumor cytotoxicity activity, thus eosinophils TCRγδ^+^ are relevant to the immune defense (164).

Regarding macrophage cellular subpopulations, several publications have reported the presence of both humans and murine TCR^+^ macrophages. The TCR-αβ was described to be expressed by peripheral blood monocytes and *in vitro* by activated monocyte-derived macrophages. Using an *in vitro* model, Beham et al. showed that TCRβ locus rearrangement and expression of Vβ repertoires in the myeloid lineage occurred during the early phase of macrophage differentiation. TCR^+^ macrophages have the ability to release CCL2 and possess high-phagocytosis capacity. These cells express molecules such as ZAP70, LAT, Fyn, and Lck, necessary for TCR signaling on lymphocytes; however, their concentration was different when macrophages received IL-4 or IFN-γ stimuli that determine different macrophages’ polarization. Interestingly, during *M. tuberculosis* infection, which derived pathology involves granuloma formation, macrophages at the inner epithelioid cell zone of caseous granulomas are TCR^+^ macrophages. Neutralization of TNF (cytokine necessary for host resistance to *M. tuberculosis*) in patients with lung tuberculosis, suppressed the expression of the CD3 ζ-subunit on TCR^+^ macrophages, becoming unstable to form the complex TCR-CD3, and the number of TCR^+^ macrophages decreased. Anti-TNF treatment is also associated with granuloma disorganization and reduced CCL2 expression, thus suggesting that TNF is a regulator of TCRαβ expression on macrophages (165).

Recently, Fuchs et al. reported that in both murine and human lesions of atherosclerosis, there is accumulation of TCRαβ^+^ macrophages. Using an *in vitro* cholesterol import and export model, this group proposed that high-density lipoprotein (HDL)-mediated cholesterol efflux induced downregulation of TCRβ within 24 h, but after 72 h, low-density lipoprotein (LDL) ingestion by macrophages-induced changes of TCRβ chains in human carotid artery lesion. Thus, cholesterol import/export was identified as a potent *in vitro* modulator of macrophages-TCRβ repertoire expression and the presence of TCRαβ^+^ macrophages was suggested as a new signature of atherosclerosis representing a novel molecular target for diagnostic and treatment of diseases where cholesterol plays a main role in physiopathology (166).

A recent report has shown that monocytes/macrophages from both humans and mice, constitutively express a second type of combinatorial receptor based on γδ variable chains, however, after a bacterial exposure, a distinct TCR Vδ repertoire is induced suggesting that TCRγδ represents a flexible host defense system that responds to bacterial challenge (167).

In conclusion, although the numbers of reports are still limited, there are evidences suggesting that the expression of TCR on cellular surface of non-lymphoid cells, including neutrophils, eosinophils, and monocytes/macrophages, specially, both TCRαβ^+^ and TCRγδ^+^ macrophages are implicated in inflammatory and infectious diseases.

## Perspectives

**Figure 3:**
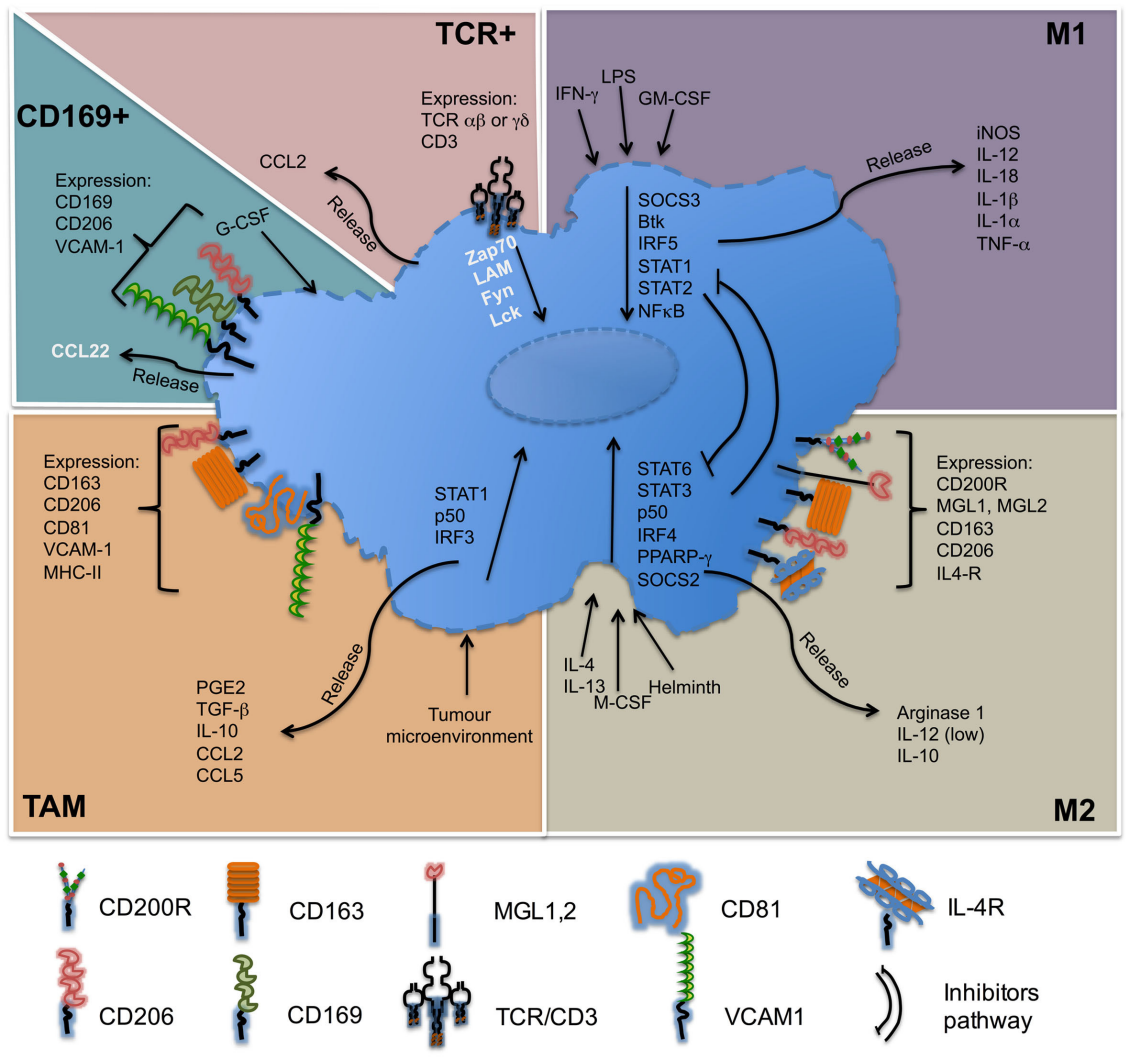
**Molecules associated with macrophage subsets**. M1 macrophages, also called classically activated, respond to stimuli such as LPS, IFN-γ, and are important producers of pro-inflammatory cytokines. M2 macrophages, also called alternatively active respond to stimuli such as IL-4 or IL-13, are producer of anti-inflammatory cytokines. Tumor-associated macrophages (TAM) respond to self-tumor microenvironment and secrete cytokines such as TGF-β or IL-10. CD169^+^ macrophages are involved in immune tolerance and erythropoiesis. TCR^+^ macrophages are a new subpopulation of macrophages that release chemokine CCL2 and play a role in inflammatory and infectious diseases. Names in black = usually described. Names in white = under specific circumstances can be present in different concentrations.

For several decades, great progress has been accomplished in the definition of lymphocyte populations by refining their phenotypic profile and characterizing their distinct functions. At present, the knowledge of macrophage subpopulations is still growing. Initially divided according to their ability to release pro-inflammatory or anti-inflammatory cytokines as a paradigm to mimic Th1 and Th2 subsets, these macrophages were designed as M1 and M2 macrophages. Nowadays, besides M1 and M2 macrophages, other different subpopulations, which have been described, such as TAM, CD169^+^, or TCR^+^, that are differently located and possess their own characteristics and functionalities. The importance of macrophages on the immune response is indubitable; they are necessary in controlling infectious processes, but their presence is also necessary to maintain homeostasis under sterile injury. In the Figure 3, we summarize some of the main features of macrophage subpopulations discussed in this review.

Many questions remain unsolved in this field, and more efforts are needed to establish origin, specific markers, conditions of appropriate stimuli for macrophage polarization, and steps of the differentiation process leading to different subpopulation of macrophages. It is still not clear the relationship between human macrophages versus animal model system and this knowledge is important to advance in the design of new therapeutic strategies supplementing those established in different pathological conditions such as cancer, infectious disease, obesity, and asthma. Moreover, preclinical and clinical observations demonstrate an association between macrophage number/type and prognosis in variety malignancies.

## Conflict of Interest Statement

The authors declare that the research was conducted in the absence of any commercial or financial relationships that could be construed as a potential conflict of interest.
